# Supplementary figures and images for: Cooperation of dual modes of cell motility promotes epithelial stress relaxation to accelerate wound healing
Source: PLoS Comput Biol. 2018 Oct 1;14(10):e1006502. doi: 10.1371/journal.pcbi.1006502 (PMC6181425; doi:10.1371/journal.pcbi.1006502)

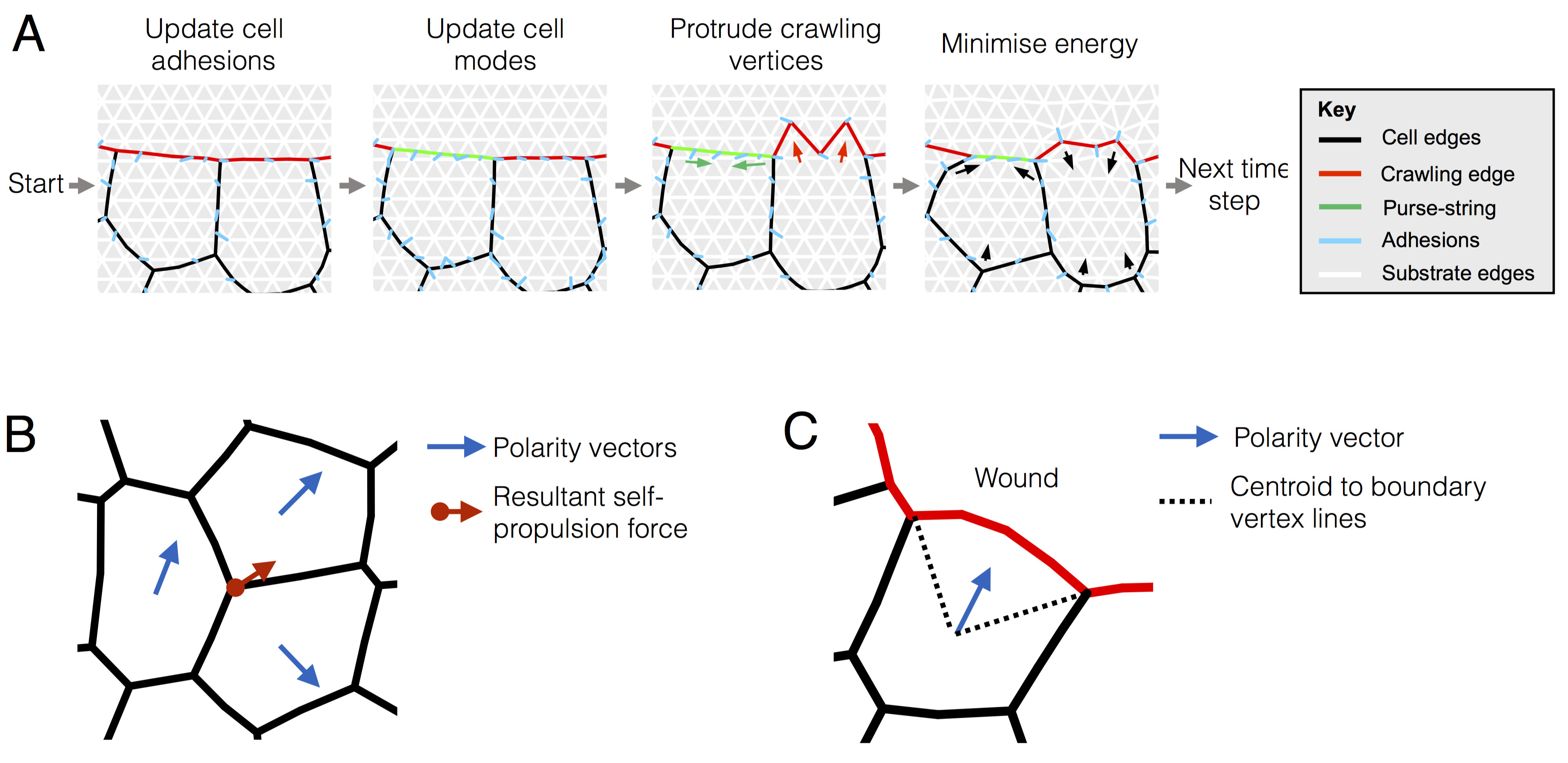

Supplement: S1 Fig — A: From left to right: 1) Update adhesion states for cell vertices. Adherent vertices attempt to unbind with a rate koff, and unbound vertices attempt to bind to the nearest substrate mesh with a rate kon. 2) Update cell modes from crawling (red) to purse-string (green) with a probability kpΔt. 3) Protrude cell edges in crawling mode (red arrows) and contract cell edges on purse-string mode (green arrows). 4) Minimize mechanical energy to move the cell vertices down their mechanical energy gradient (black arrows). B: Illustration of self-propulsion force on a vertex in the bulk. The central vertex has a resultant force (red arrow) equal to the average force from its adjacent cells (blue arrows). C: The polarity vector (blue arrow) for a cell around the wounds bisects the angle between the lines from the cell centroid to the boundary vertices (dashed lines). (TIF) [file pcbi.1006502.s001.tif]

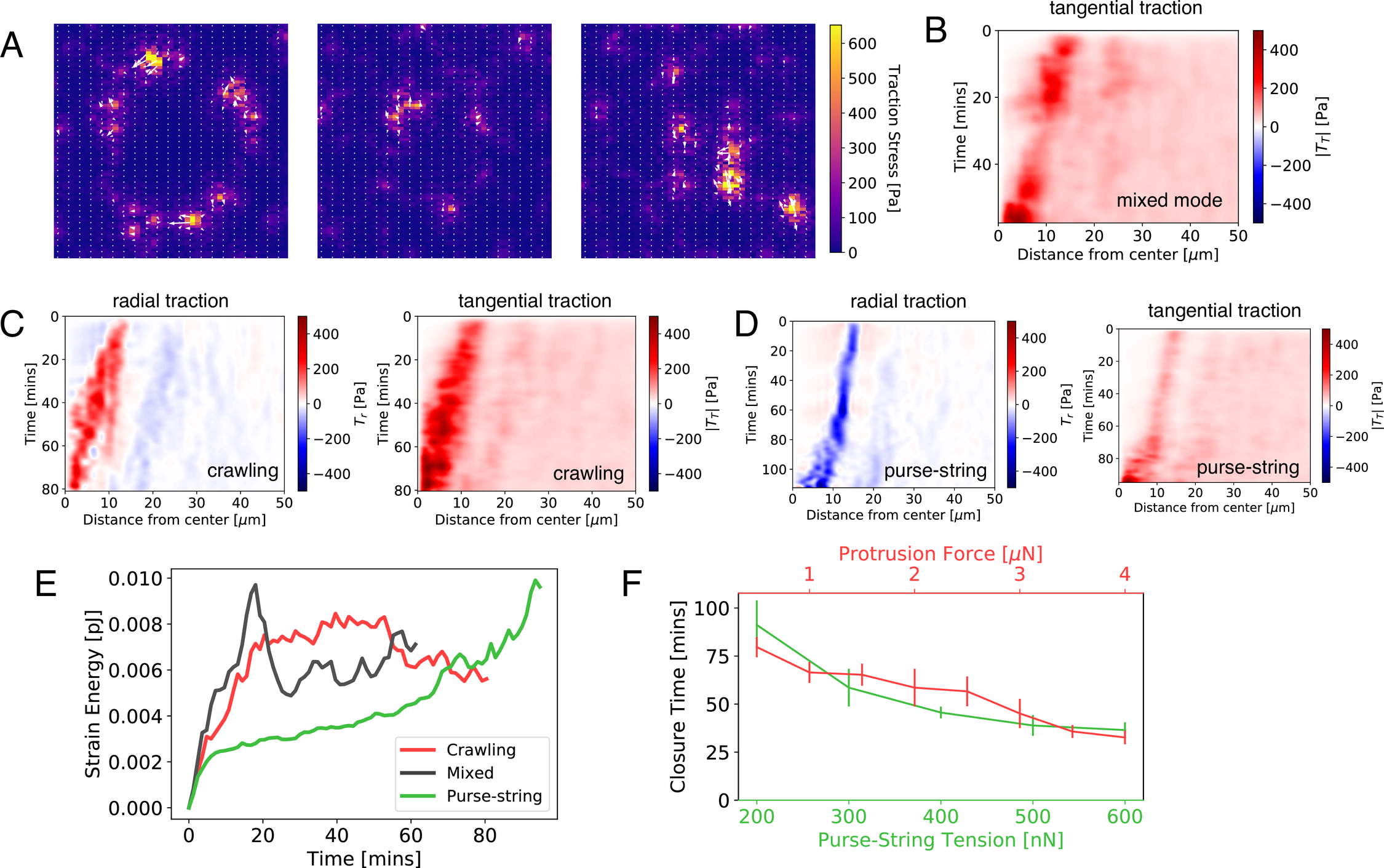

Supplement: S2 Fig — A: Traction stress distribution around a closing wound with kp = 4 hr−1, at t = 5 min (left), t = 30 min (middle), t = 60 min (right). B: Kymograph of tangential traction stress for the mixed mode of closure (kp = 4 hr−1). C: Kymographs of radial and tangential traction stress for the crawling (kp = 0 hr−1) mode of closure. D: Kymographs of radial and tangential traction stress for the purse-string (kp = 1000 hr−1) mode of closure. E: Total strain energy transmitted vs time for crawling, purse-string, and mixed modes of closure. F: Closure time as a function of purse-string tension (green) and protrusion force (red) for a mixed mode of closure (kp = 4 hr−1). Error bars represent standard error of mean. (TIF) [file pcbi.1006502.s002.tif]

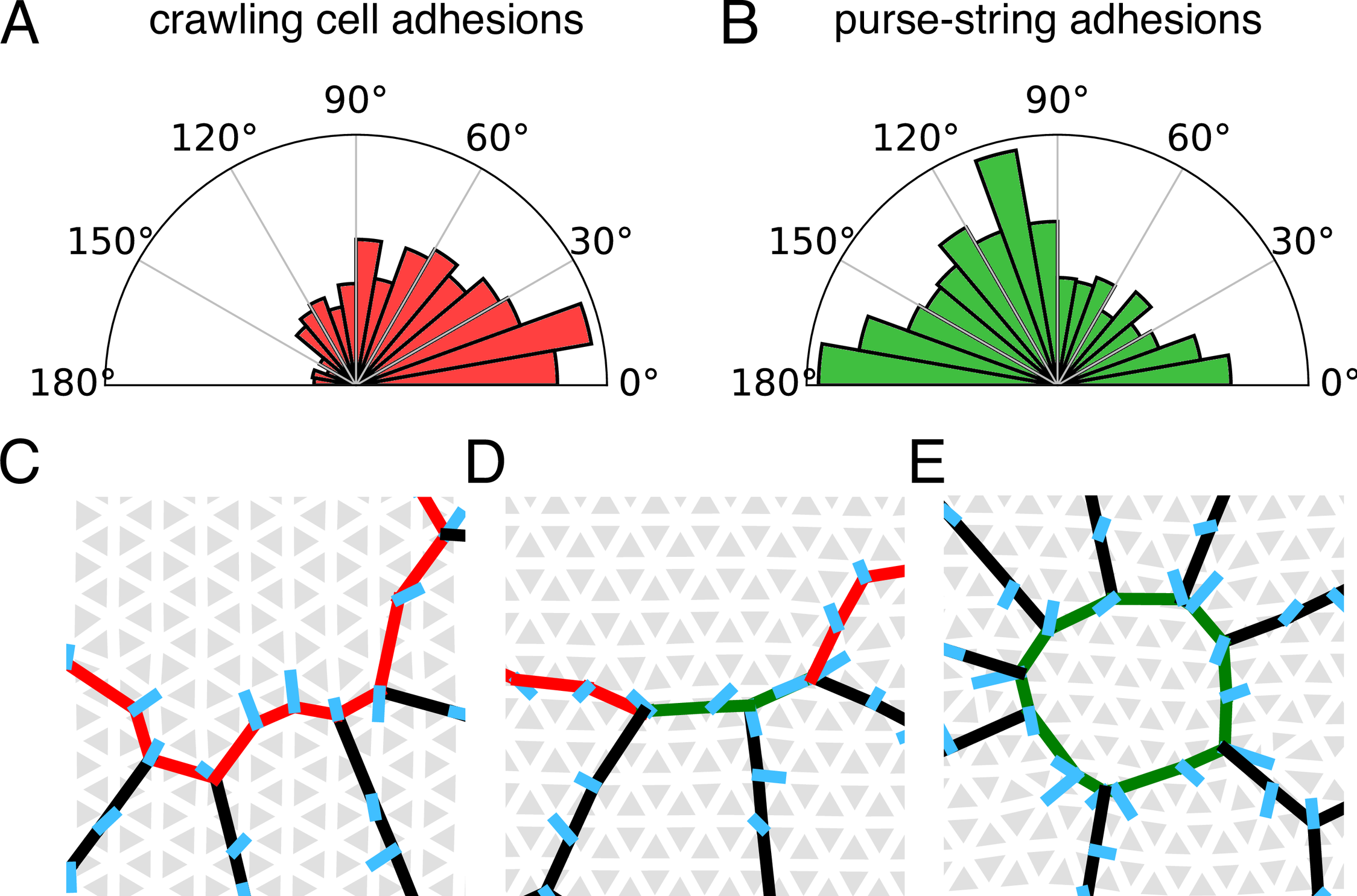

Supplement: S3 Fig — Histograms of the local angle between cell-substrate bonds and the radial vector to the wound center, in (A) crawling and (B) purse-string cells at the leading edge. (C) Representative image of crawling cells with focal adhesions oriented normal to the wound edge. (D) A purse-string edge flanked between two crawling edges have its focal adhesions parallel to the wound edge. (E) Purse-string only wounds have a majority of adhesions oriented normal to the wound edge, due to normal driving forces arising from contractile tension in the purse-string. Green segments represent purse-string edges, while red segments are crawling cells. (TIF) [file pcbi.1006502.s003.tif]

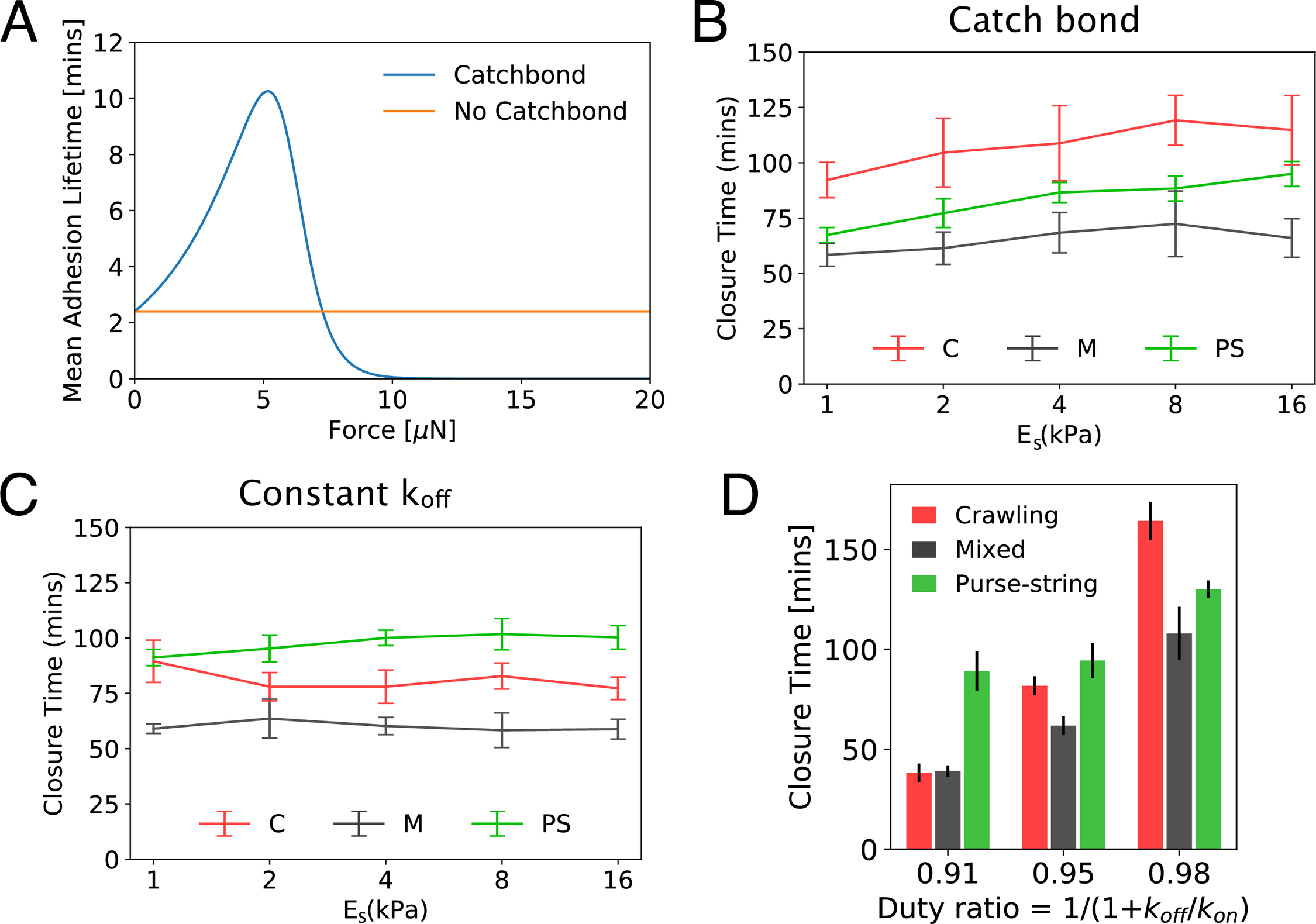

Supplement: S4 Fig — (A) Mean adhesion lifetime, koff-1, vs applied force for a catch-bond model (blue) and constant koff (yellow). (B) Substrate stiffness dependence of wound closure time for a catch-bond model of cell-substrate adhesions, for crawling (red), purse-string (green) and mixed (kp = 4 hr−1, black) modes of closure. (C) Wound closure time vs substrate stiffness for constant koff. Each data point represent average over 6 simulations. Error bars show standard deviation. (D) Closure time vs duty ratio of focal adhesion bonds, kon/(koff + kon), for crawling, purse-string and mixed modes of wound closure. Duty ratio is varied by changing the detachment rate, koff, for a fixed kon. (TIF) [file pcbi.1006502.s004.tif]

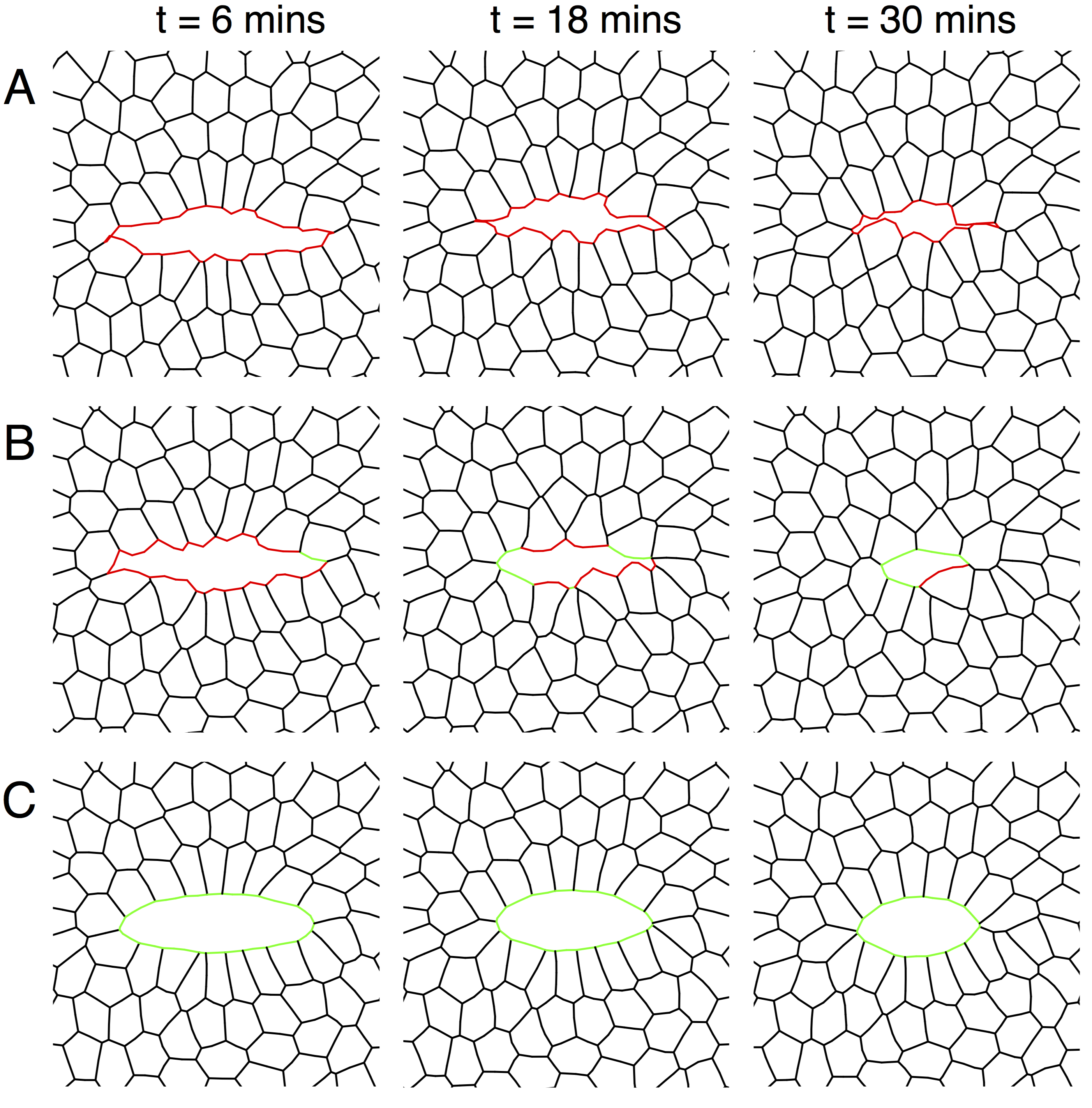

Supplement: S5 Fig — Wound morphologies for (A) crawling (kp = 0 hr−1), (B) mixed (kp = 4 hr−1), and (C) purse-string (kp = 1000 hr−1) modes of closure, at t = 6 min (left), t = 18 min (middle), t = 30 min (right). The initial aspect ratio of the wound is 4. (TIF) [file pcbi.1006502.s005.tif]

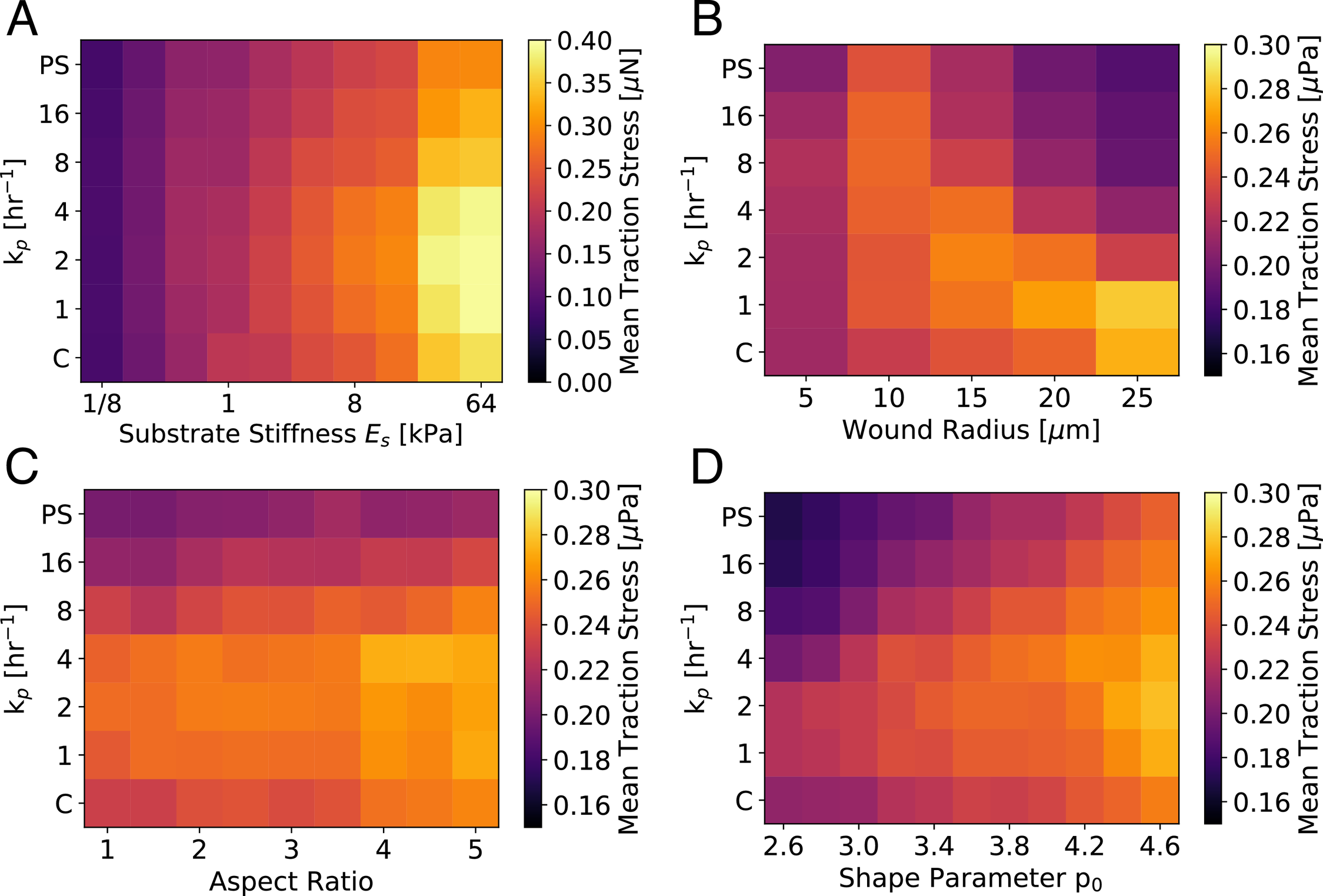

Supplement: S6 Fig — Temporal mean of spatially averaged traction stress during wound closure for different values of kp and (A) substrate stiffness, (B) wound radius, (C) wound aspect ratio, and (D) shape parameter p0. (TIF) [file pcbi.1006502.s006.tif]

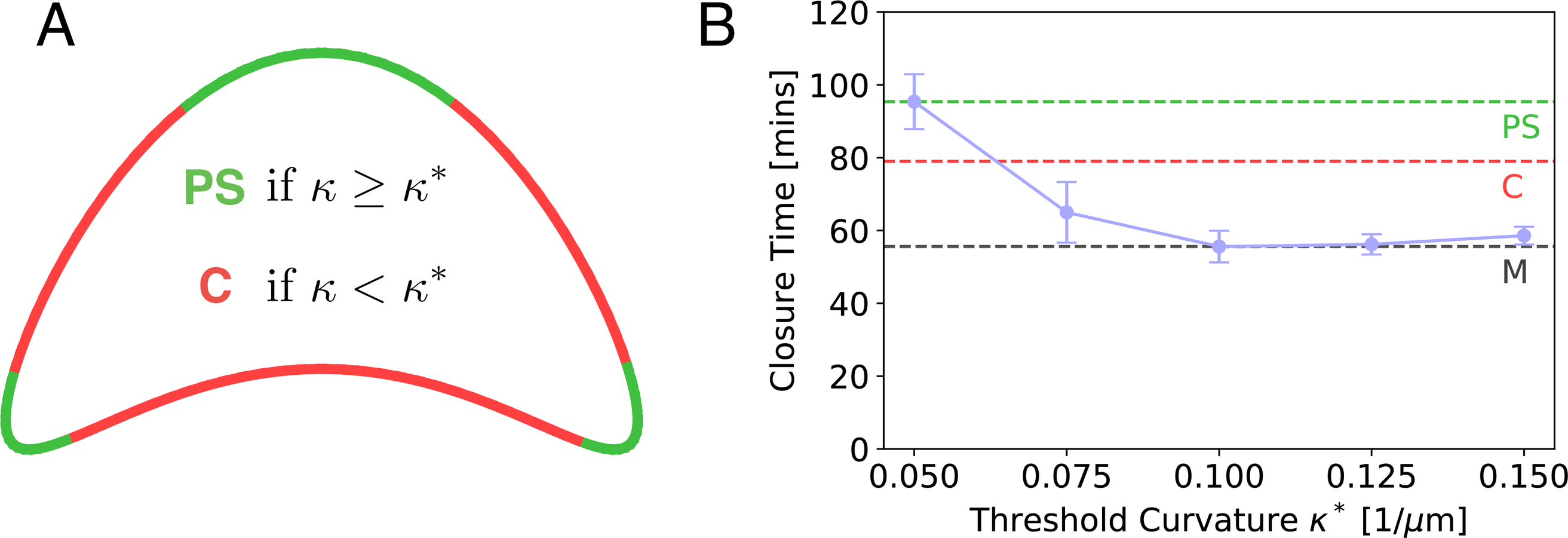

Supplement: S7 Fig — (A) Schematic showing purse-string and crawling edges for a wound with non-uniform curvature. Purse-string (PS; green) forms on leading edges with curvature κ > κ*, where κ* is a threshold curvature. Cells prefer to crawl (C; red) if κ < κ*. (B) Wound closure time vs κ* for the concave shaped wound in (A). The optimum threshold curvature is chosen to be the one that minimizes wound closure time. Dashed lines indicate wound closure times for pure crawling (red), pure purse-string (red) and stochastic mixed (black) modes of closure. (TIF) [file pcbi.1006502.s007.tif]

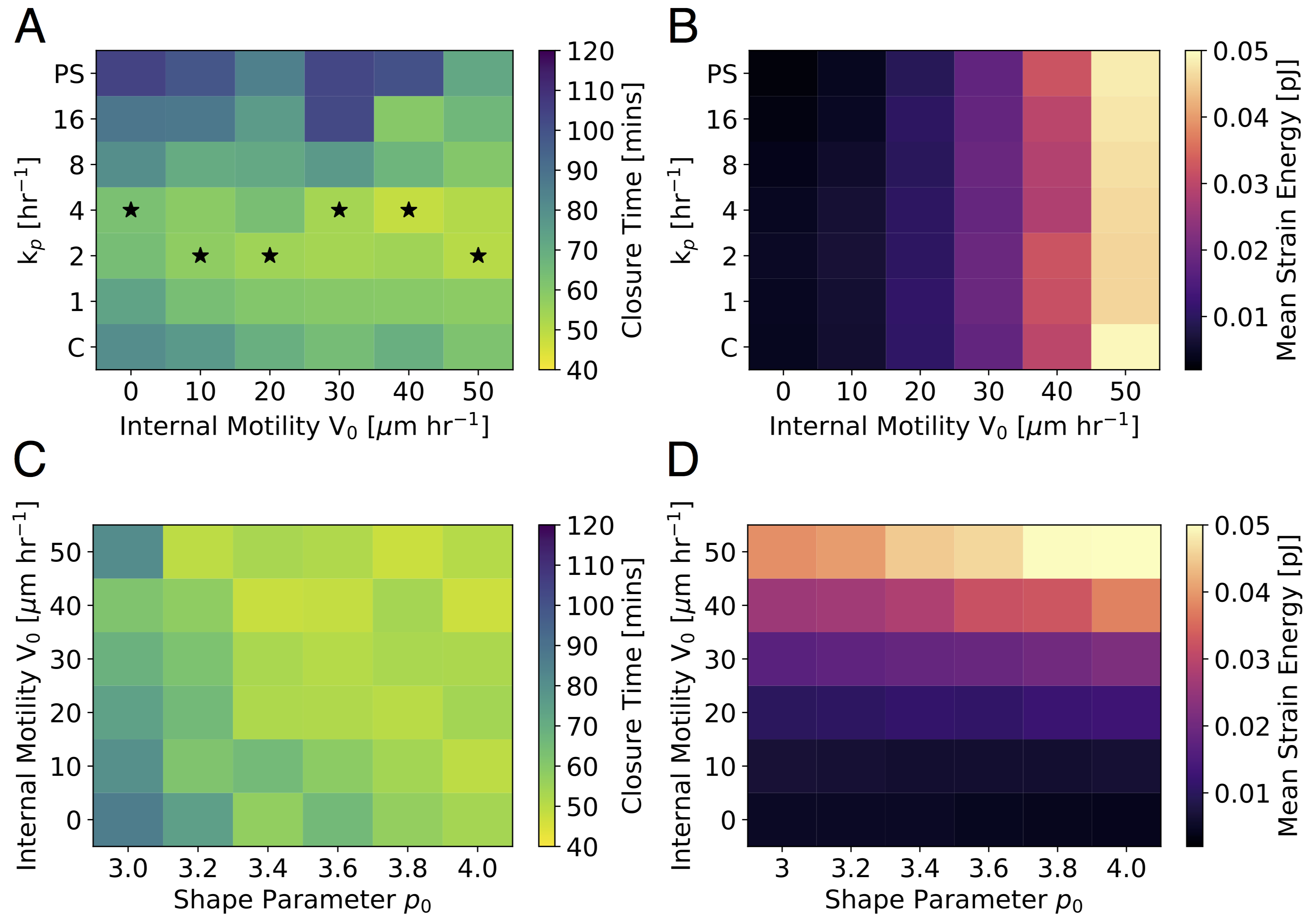

Supplement: S8 Fig — (A) Closure time, and (B) mean strain energy for different values of internal motility v0 and kp. Starred cells indicate the fastest wound closure for a given v0 with varying purse-string assembly rates. (C) Closure time, and (D) average strain energy for different values of shape parameter p0 and internal motility v0, for a mixed mode of closure (kp = 4 hr−1). (TIF) [file pcbi.1006502.s008.tif]

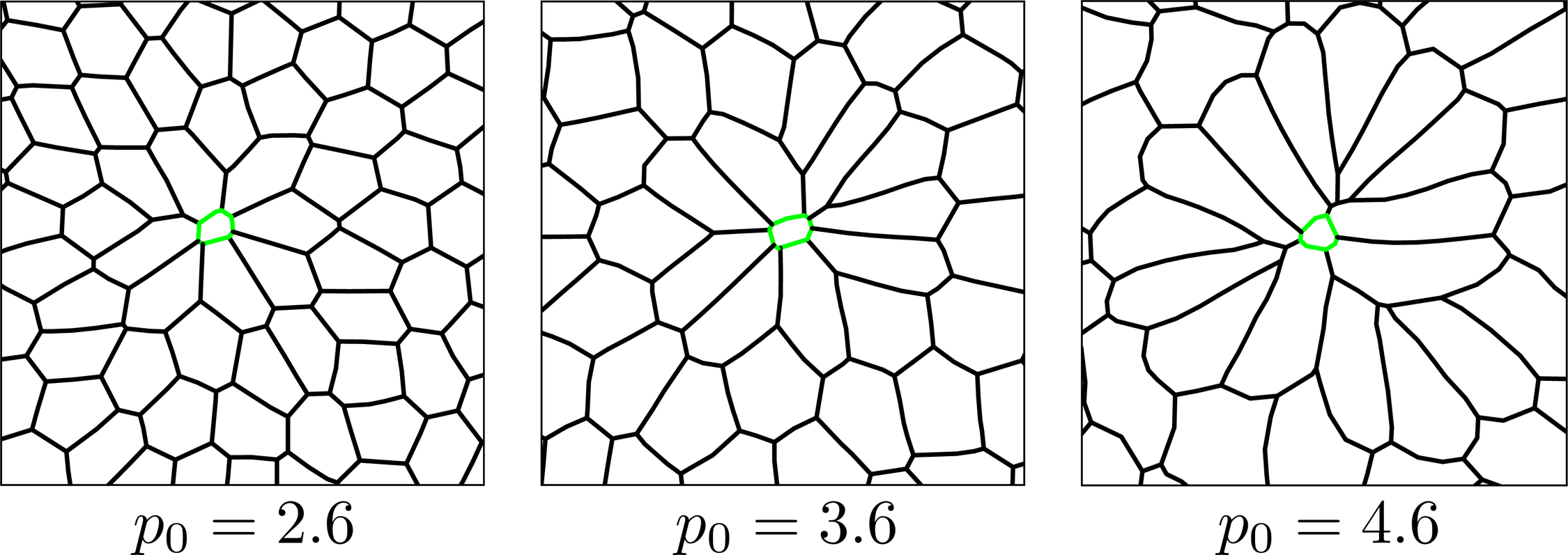

Supplement: S9 Fig — (TIF) [file pcbi.1006502.s009.tif]

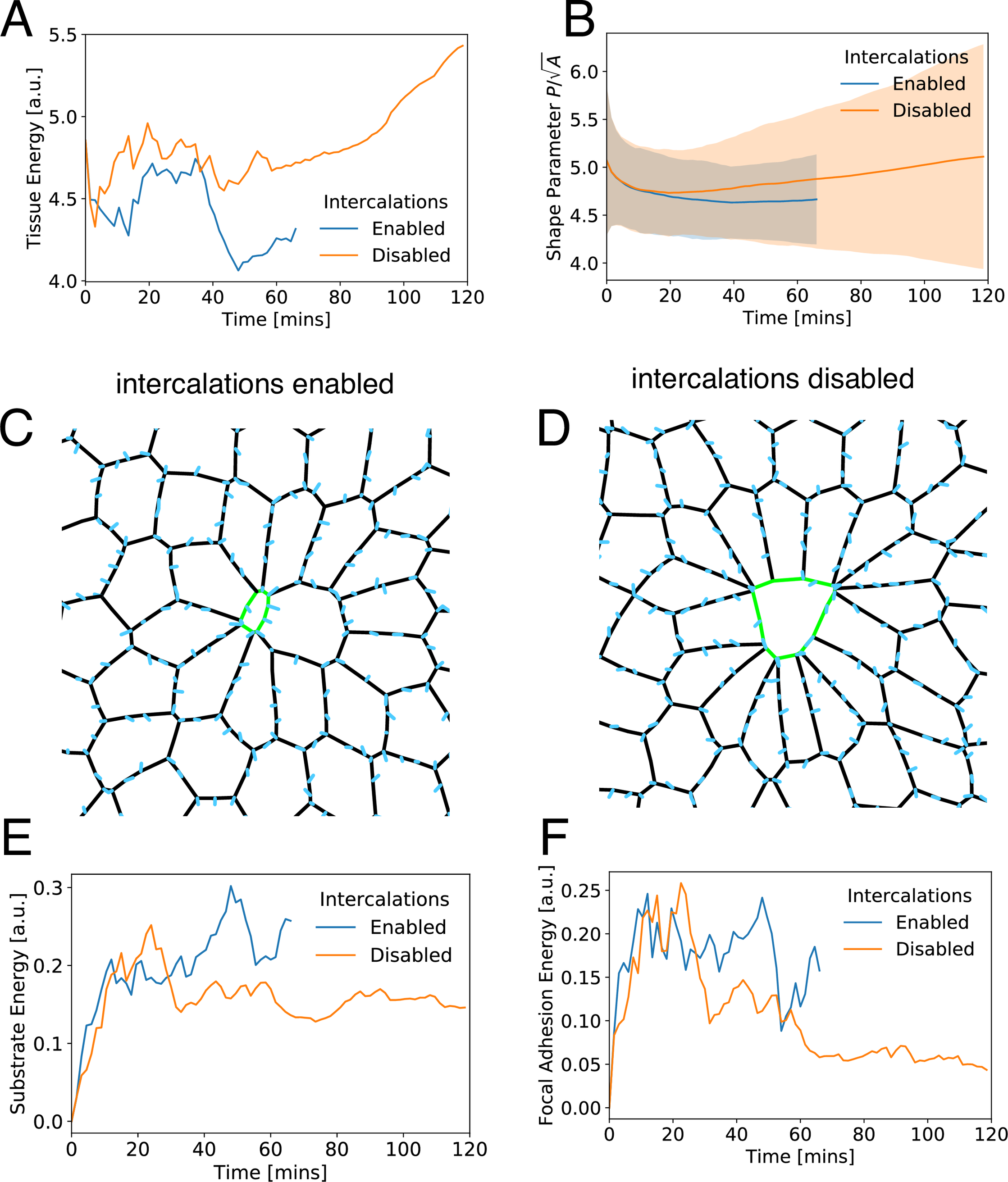

Supplement: S10 Fig — (A) Total tissue mechanical energy vs time, with intercalations enabled and disabled during wound closure. (B) Mean cell shape parameter vs time. Shaded regions represent one standard deviation. With intercalations disabled, cells elongate and have more variability in shape. (C-D) Simulation image showing tissue morphology before closure with intercalations (C), and in a jammed state without intercalations (D). Cells are much more elongated when intercalations are disabled. (E) Total substrate strain energy, and (F) total focal adhesion strain energy, over time with intercalations enabled and disabled. (TIF) [file pcbi.1006502.s010.tif]

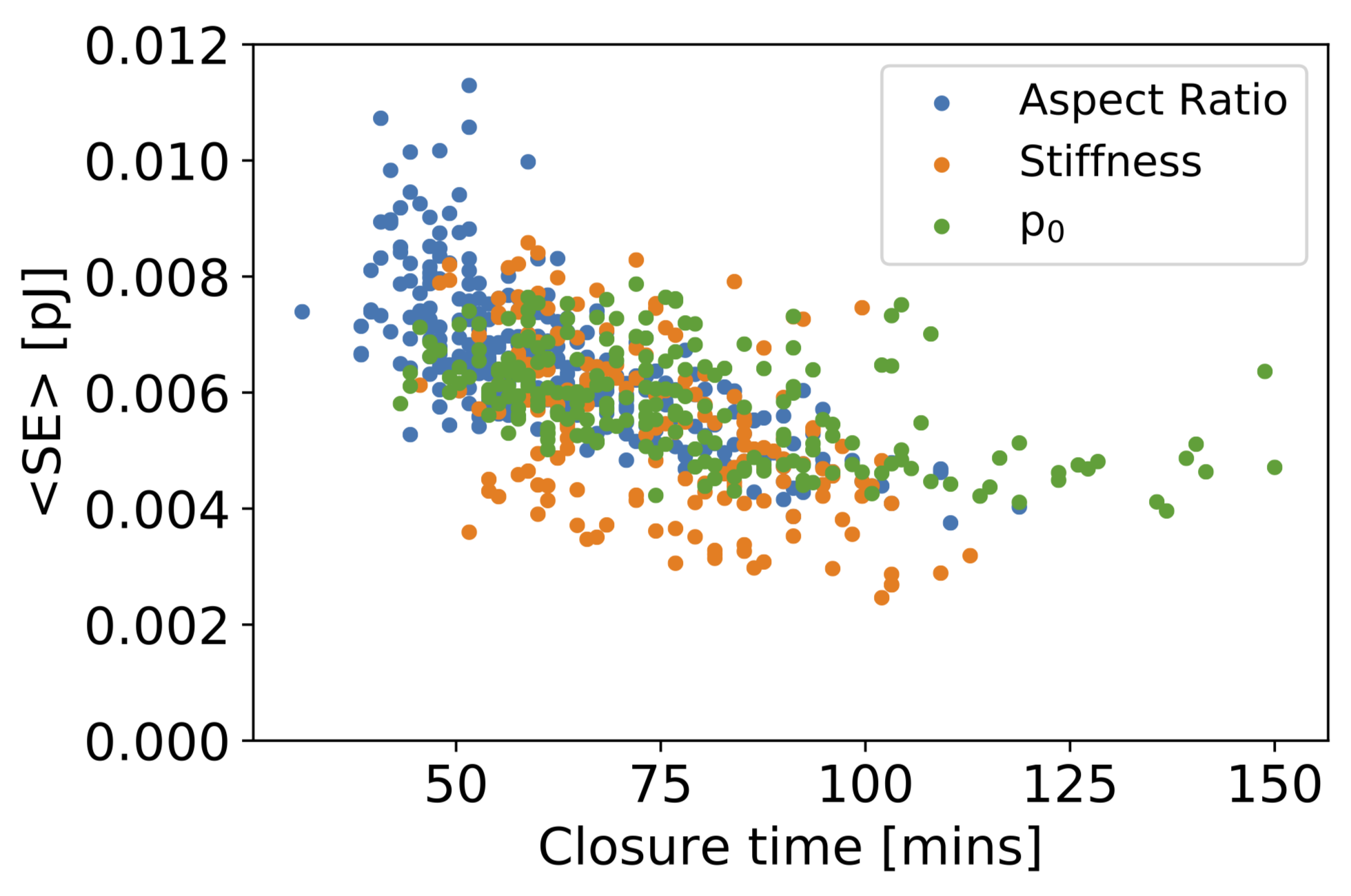

Supplement: S11 Fig — Mean strain energy vs closure time. Each data point represents a different simulation. The color corresponds to the parameter that was being varied in that simulation. (TIF) [file pcbi.1006502.s011.tif]

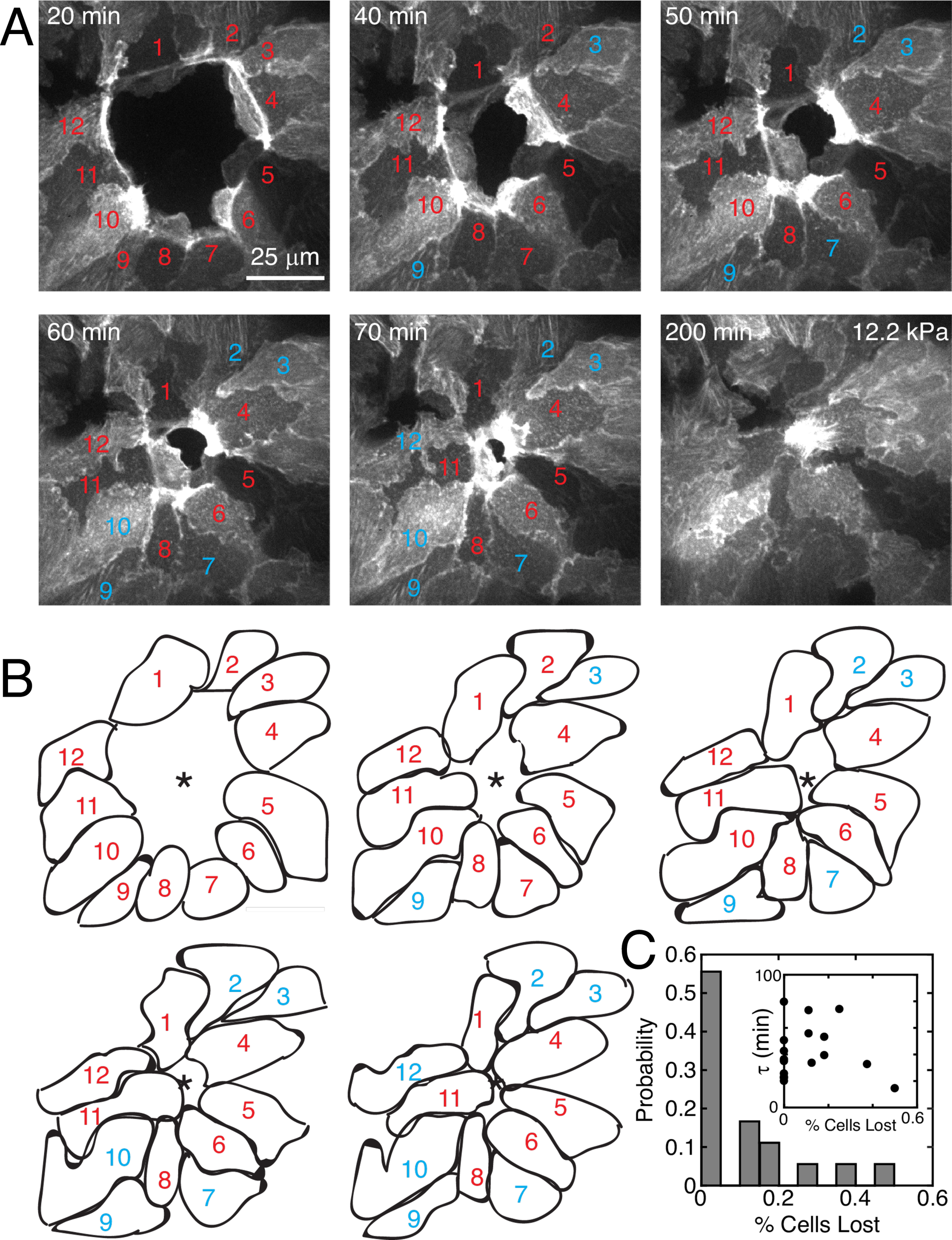

Supplement: S12 Fig — (A) Time-lapse images of fluorescent F-actin within MDCK cells closing a wound and (B) the drawn outlines of cells initially at the leading edge. Cells at the leading edge at each time point are numbered in red, whereas cells excluded from the leading edge during closure are numbered in cyan. (C) The probability distribution of fractional cell loss for N = 18 wounds where the average number of cells initially at the leading edge is 9 ± 2. N = 8 wounds exhibit a loss of cells at the leading edge during closure. Within this subset, the average percentage of cells lost is 0.23 ± 0.14. (C-inset) The closure timescale, τ, calculated from A(t) = A(0)e−t/τ, where A(t) is the area of the wound at time t, vs the fractional cell loss at the leading edge. (TIF) [file pcbi.1006502.s012.tif]

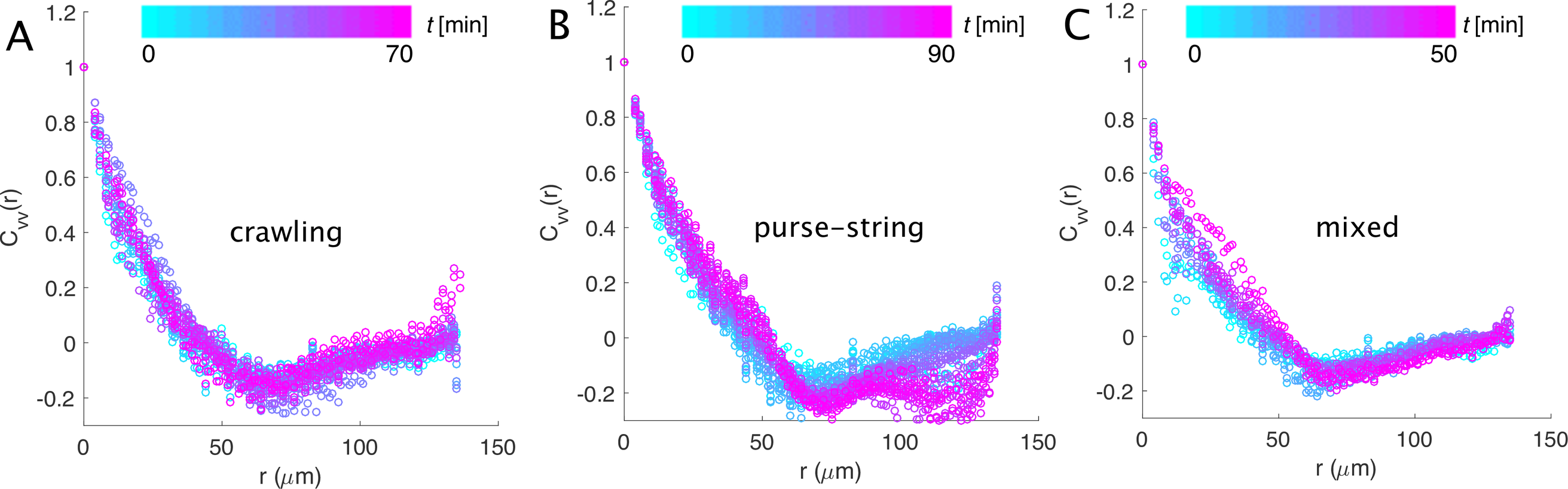

Supplement: S13 Fig — Figure shows velocity-velocity correlation function, Cvv(r) = 〈v(0)·v(r)〉/〈v(0)2〉, where r is the distance between two cell center velocity vectors, v. Cvv(r) is shown at different time points (indicated by color) for (A) crawling, (B) purse-string, and (C) mixed modes of wound closure. Velocity vectors of cells on opposite sides of the wound are anti-correlated. (TIF) [file pcbi.1006502.s013.tif]

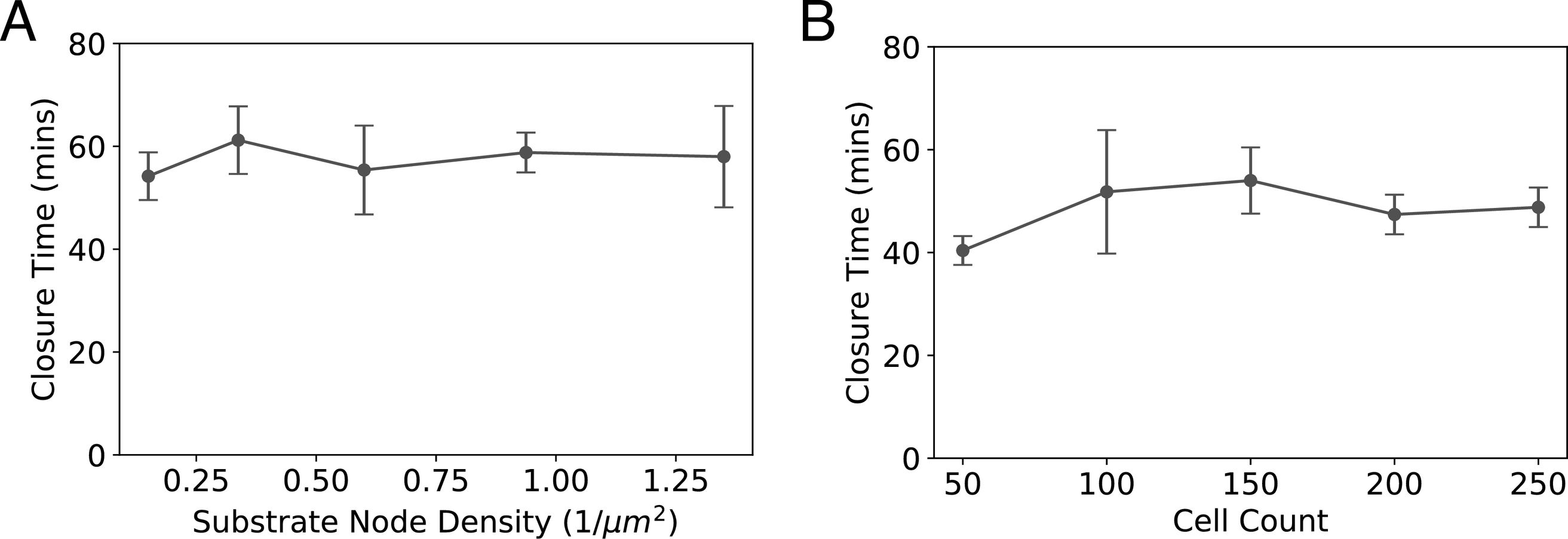

Supplement: S14 Fig — Closure time vs (A) cell count, (B) density of nodes in the substrate spring mesh, for a wound of fixed initial size. (TIF) [file pcbi.1006502.s014.tif]

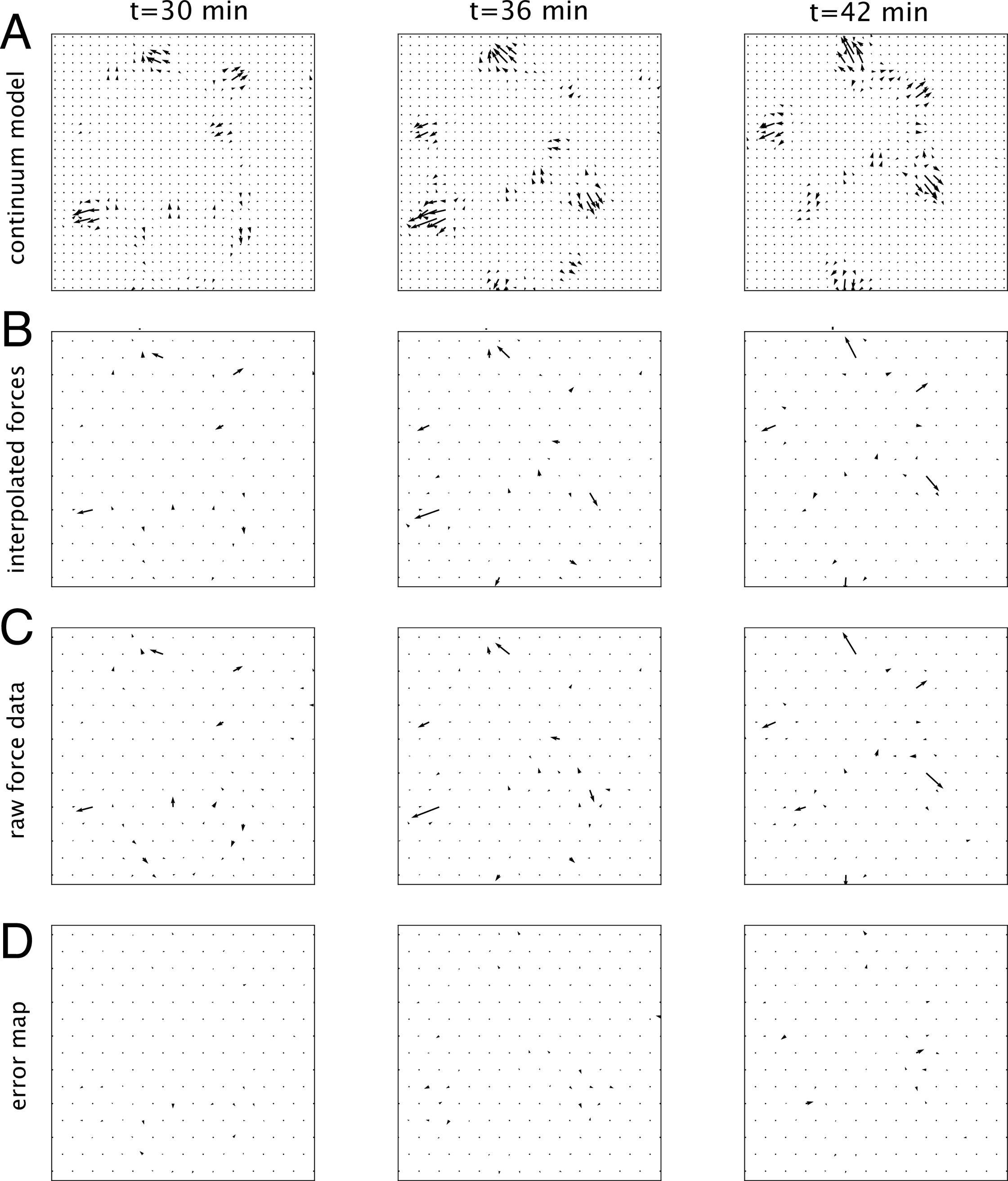

Supplement: S15 Fig — Figure shows traction force vectors using two different methods computed during wound closure at t = 30 min (left column), t = 36 min (middle column), and t = 42 min (right column). (A) Traction force vectors computed using the continuum elasticity Eq (9). (B) Continuum model based forces in (A) interpolated on the substrate triangular mesh. (C) Traction forces directly computed from displacements in the substrate spring mesh. (D) Error map showing the difference of traction force vectors in (B) and (C). Lengths of arrows are proportional to the magnitude of the traction force, and the scale is consistent between images. (TIF) [file pcbi.1006502.s015.tif]
